# A Simple Semi-Automatic Approach for Land Cover Classification from Multispectral Remote Sensing Imagery

**DOI:** 10.1371/journal.pone.0045889

**Published:** 2012-09-26

**Authors:** Dong Jiang, Yaohuan Huang, Dafang Zhuang, Yunqiang Zhu, Xinliang Xu, Hongyan Ren

**Affiliations:** State Key Lab of Resources and Environmental Information System, Institute of Geographical Sciences and Natural Resources Research, Chinese Academy of Sciences, Beijing, China; University of Oxford, United Kingdom

## Abstract

Land cover data represent a fundamental data source for various types of scientific research. The classification of land cover based on satellite data is a challenging task, and an efficient classification method is needed. In this study, an automatic scheme is proposed for the classification of land use using multispectral remote sensing images based on change detection and a semi-supervised classifier. The satellite image can be automatically classified using only the prior land cover map and existing images; therefore human involvement is reduced to a minimum, ensuring the operability of the method. The method was tested in the Qingpu District of Shanghai, China. Using Environment Satellite 1(HJ-1) images of 2009 with 30 m spatial resolution, the areas were classified into five main types of land cover based on previous land cover data and spectral features. The results agreed on validation of land cover maps well with a Kappa value of 0.79 and statistical area biases in proportion less than 6%. This study proposed a simple semi-automatic approach for land cover classification by using prior maps with satisfied accuracy, which integrated the accuracy of visual interpretation and performance of automatic classification methods. The method can be used for land cover mapping in areas lacking ground reference information or identifying rapid variation of land cover regions (such as rapid urbanization) with convenience.

## Introduction

Land use and land cover change (LUCC) has increasingly become a central component of global environmental change research [1], [2]. Land cover data are the basis of this research and are key input to earth surface processes, including bio-chemical cycles, spatial and temporal distribution of plant biomass and respiration, and coupling between the atmosphere and biosphere [3]. Satellite imagery has been the primary tool for large-scale time-dependent land cover mapping [4]. Numerous classification algorithms utilize pattern recognition techniques, spectral features of the images and empirical geographical knowledge for land use mapping. Recent efforts to map land cover using remote sensing data have taken a variety of approaches, including visual interpretation classification [5], unsupervised clustering coupled with extensive ancillary data and manual labeling of clusters [6], supervised classification [7], expert system classification [8], artificial intelligence neural network classification [9], and decision tree classification [10], [11]. Traditional unsupervised classification algorithms, such as maximum likelihood classification, use clustering techniques to identify spectrally distinct groups of data and are the earliest approach of land cover automatic classification that has employed pattern recognition techniques. The drawback of these algorithms is that the accuracy of land cover classification is not guaranteed and the land cover classifications are arbitrary. Supervised classification methods require substantial expertise and human participation for selecting training samples. Therefore, the result of land cover classification is influenced greatly by classification participants, and it is impossible to classify land cover automatically with these methods. Furthermore, the algorithms such as neural network classification and fuzzy logic classification are highly complicated in their algorithm basis which makes them difficult to understand and apply widely. Decision tree classification methods are widely used in large areas, such as global land cover mapping (e.g. land cover type Yearly 1 km GRID of EOS/MODIS [12]). The main problem presented by decision tree classification is the construction of the decision tree and the assignment of thresholds for each sub nodes, which heavily depends on human experience and varies spatially and temporally.

To improve the accuracy of classification, images taken at multiple times were adopted for change detection. Numerous algorithms have been proposed for identifying and analyzing pixel-by-pixel differences between images of the same area acquired at different times [13], most of which produce reliable change detection results. Although the understanding of the causes of land use change have improved over the last several decades [14], accurate identification of the land cover types of changed pixels based on comparing distinction between two images without validation data is notably difficult. It is because the two images under consideration are often from slightly different times of the year, which may cause errors in change detection. To overcome problems of change detection, Fortier *et al.* proposed a new technique for land classification of imagery lack of ground data [15], which used temporally invariant ground features as calibration and validation data. It provided an innovative and accurate methodology for land cover classification using remote sensing imagery for which ground data are not available. However, manual steps of invariant features identification and classification tree algorithm (CTA) used for image classification made the methodology require human participation and hard to classify images automatically.

In light of the afore-mentioned problems, we propose a simple but robust method for land cover classification using satellite images. Prior accurate land cover data are adopted as important background information. Using only the prior land cover map, a recent image was classified. Our approach can be simply described to two steps: (1) semi-automatically detecting land cover changed pixels from satellite images compared with prior land cover map; (2) semi-automatically classifying the land cover of changed pixels based on pattern recognition and changed rules. This method was evaluated for the Qingpu District in Shanghai, China with images from HJ-1 Environment Satellites (HJ-1), which is a new type of satellite developed by China and launched in 2008, being used for environment and disaster monitoring.

## Methods

**Figure 1:**
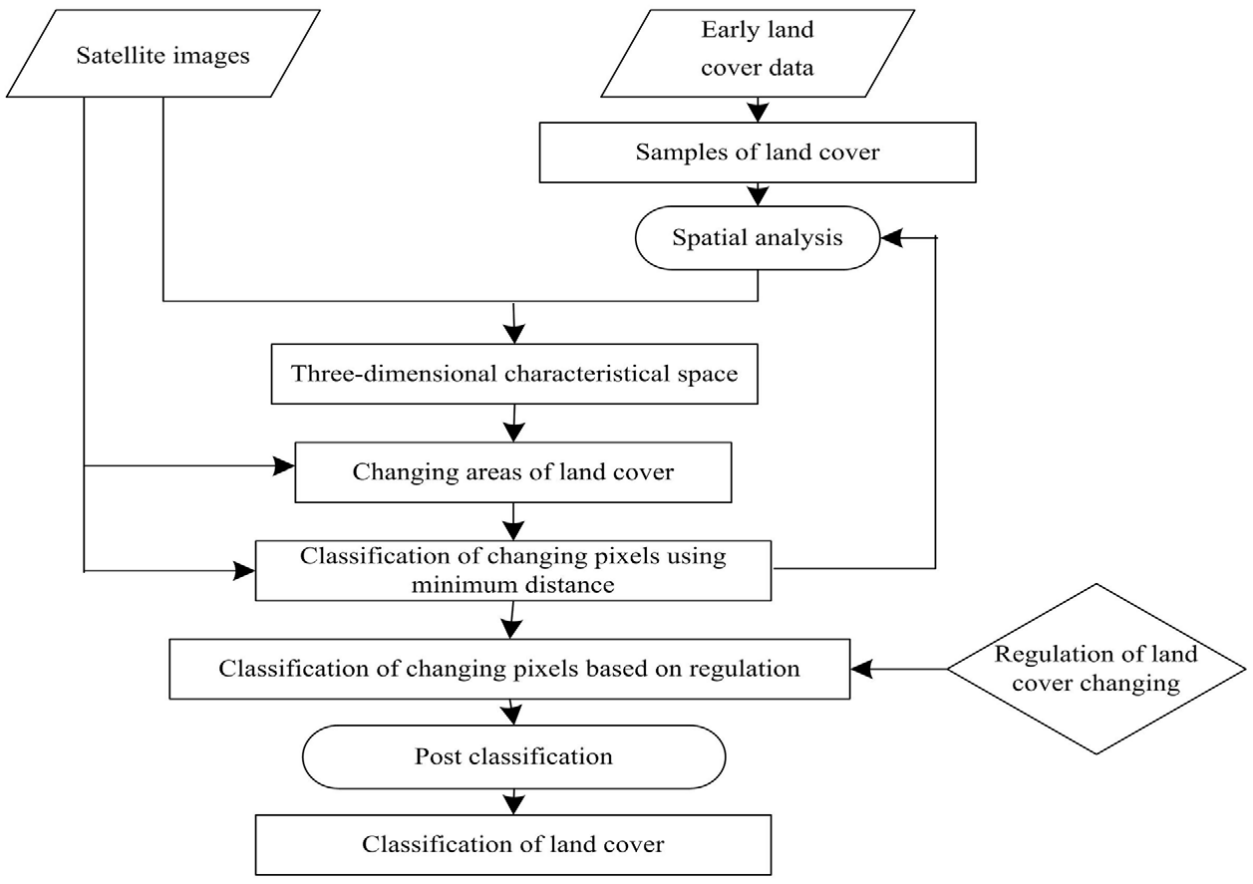
Flowchart of proposed land cover classification method.

The main phases for implementation of the new method are shown in Fig. 1.

### Automatic Collection of Training Samples

Similar to supervised classification, the first step of our method is obtaining training samples with the purpose of obtaining pure pixels for various land cover classes. Previously, accurate sampling depended entirely on human participation and interpretation, which reduces the automation of land cover classification. In this paper, we propose a new method to extract pure pixels of land cover automatically with an accurate previous land cover dataset as prior knowledge. The 1∶100,000 vector land cover maps of China produced by the Chinese Academy of Sciences were used in our study. This database has been validated by intensive field surveys, including a total survey length of 75,271 km across China. The overall accuracy of the land use map is 95% for all land use classes, which is the best map available at the national scale for China [16]–[19].

Ecologically, the junctions of different land cover classes are fragile areas, where are the main areas of land cover changing. We assumed that the interiors of individual land cover areas are relative ecologically stabile areas and that larger patches are always more ecologically stabile. Based on this assumption, the patches of land covers were sorted in descending order of their area. Samples of different land covers were selected based on their accumulation area threshold (*Pa*), the condition of land cover classification that can be defined as follows:
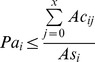
(1)where 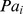 is the accumulation area threshold of the *i*th class of land cover, 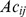 is the area of patches of the *i*th class of land cover sorted in descending order, 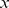 is the number of the *i*th class of land cover, and 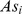 is the total area of the *i*th class of land cover.

**Figure 2:**
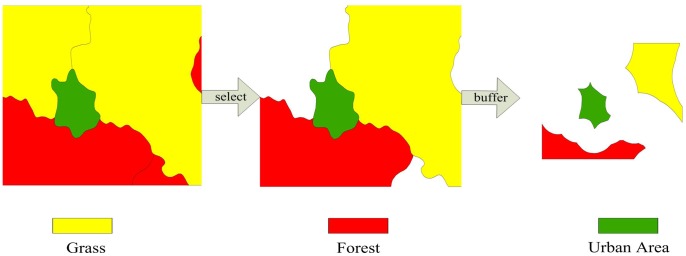
Sketch map of automatic collection of training samples.

We discarded the joint region of different land cover with spatial buffer analysis. The patch areas vary for land cover data; therefore, the buffer area cannot be obtained using the same distance to all patches. The distance of buffer analysis is
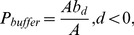
(2)where 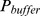 is area threshold for buffer analysis, 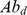 is the buffer area of the patch with a distance of *d*, with *d* negative, and *A* is the area of the patch. The buffer regions were collected as training samples, and the automatic collection of training samples is illustrated in Fig. 2.

To keep the precision and efficiency of the method we proposed, it is necessary to determine an optimal value of 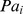 and 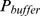. Through a series of experiments using data from 4 test areas, including the area of Qingpu which will be used as evaluating area, we determined the (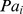,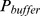) combination of (60%, 50% to 60%) to be optimal for samples of land cover classification selection.

### Establishment of Three-dimensional Feature Space

The satellite image for classification was first overlaid with a previous land cover map. Multispectral values of each pixel were extracted based on the prior land cover type. Principal component statistical analysis was processed for the data in all spectral bands of each land cover class extracted from the region of interest. Based on previous studies [20]–[22], we selected the first three principal components for orthogonal decomposition to construct the three-dimensional feature space of different land cover classes:
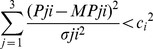
(3)where *i* is the *i*th class of land cover, 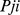 is the *j*th principal component of the *i*th class of land cover, 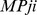 is the mean value of the *j*th principal component of the *i*th class of land cover, 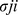 is the standard deviation of the *j*th principal component of the *i*th class of land cover, and 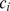 is the ellipsoid radius of the three-dimensional feature space of the *i*th class of land cover.

The three-dimensional feature space of different land cover classes is later used for the detection and classification of changed land cover pixels in the following steps.

### Change Detection

The detection of changed areas of land cover is a key step in our method. During this step, the satellite images were overlaid with early land cover maps, and the spectral data of the images were extracted according to different land cover classes of early land cover maps. For each land cover class, all extracted cell spectral data of that class were applied to equation 2 to calculate the values of the corresponding feature space. The pixels outside of the corresponding three-dimensional feature space were considered to be the changed areas, which is similar to assignment of thresholds for sub nodes in decision tree classification methods.

In equation 2, 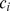 is determined in this step, for the changed rates of different land cover classes are different. The pixels were calculated iteratively until the proportion of changed areas to the total area of the corresponding land cover class was within a reasonable range. The proportional area of each changed land use cover class was determined with the aid of expert knowledge of the census and other monitor value change in the study area, such as population, gross domestic product (GDP), crops yield and so on. The ellipsoid radius of the three-dimensional feature space of 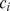 increased with the iterative calculation until this requirement was met.

### Classification of Changed Pixels

After obtaining the changed land cover pixels, the satellites images and three-dimensional feature space were used to classify them based on pattern recognition and changed rules.

The initial classification of the changed area of land cover was determined by calculating the minimum spectral distance based on the three-dimensional feature space. For each changed pixel, the spectral data of all bands were input to the formula for all land cover classes in three-dimensional feature space to calculate the minimum spectral distance (*d_min*):
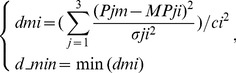
(4)where 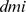 is the three-dimensional spectral Euclidean distance of the *m*th changed pixel to the *i*th class of land cover based on the feature space, 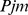 is the *j*th principal component of the *m*th changed pixel in corresponding feature space of the *i*th land cover class, 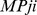 is the mean value of the jth principal component of the *i*th land cover class, and 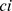 is the ellipsoidal radius of the three-dimensional feature space of the *i*th class of land cover. The initial classification of changed pixels was determined by the minimum value of the distance, 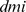.

The changing rule of land cover classification was adopted to revise the initial classification result. Previous knowledge of the land cover change could be used in this phase. In several agricultural regions, farmland is protected by a national policy that aims to ensure at least 120,000 km^2^ of farmland in China. In other regions with pronounced urbanization, urban areas are expanding rapidly and are not changed into other land cover classes, such as farmland or woodland. In this study, a drag coefficient of changed land cover (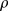) is introduced to express the rules of changed land cover. For all *n* types of land cover classes, 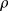 is an n-by-n matrix. The final land cover classification of changed pixels were determined by combination of 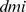 and 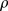. The minimum distance of the land cover classification based on changed rules is defined as:
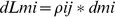
(5)where 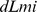 is the minimum distance of the *m*th pixel to the *i*th land cover class based on the changed rules and 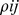 is the drag coefficient of the *i*th land cover class that changed to the *j*th land cover class.

### Modification of Post-classification Results

Classified images often suffer from a lack of spatial coherency (spotting or holes in classified areas). Low-pass filtering could be used to smooth these images, but the class information would be contaminated by adjacent class codes. This problem is solved by modification of the post-classification results. The land cover classes are clumped together by first performing a dilation operation and later an erosion operation on the classified image [23], [24]. In this study, any group of pixels, which was less than 3 pixels in size, was identified as noise in dilation operation. A 3-by-3-pixel moving window was used to eliminate noise and remove salt-and-pepper effects.

## Results and Discussion

### Study Area and Data

#### Study area

**Figure 3:**
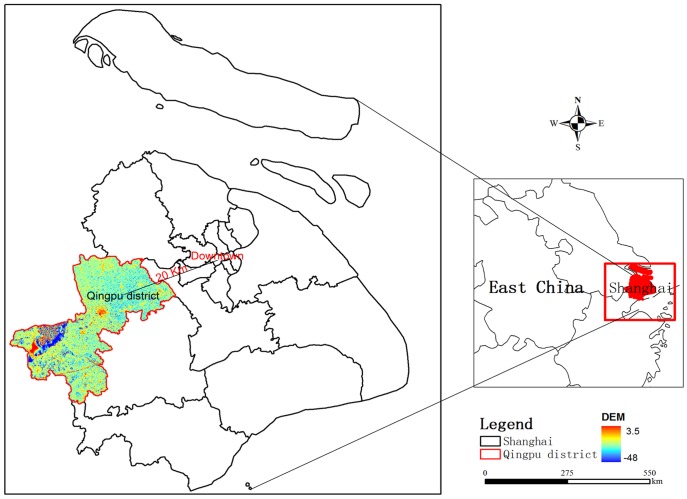
Location of the study area: Qingpu District, Shanghai, China.

The study area of Qingpu district of Shanghai, China covers 676 *km^2^* and encompasses over 10% of Shanghai city (Fig. 3). Approximately 60% of study area is dominated by annually double crops land, mainly growing rice and wheat. The rest portion of the study area is largely composed of two land covers which are residential and build-up land and water [25]. The study area located in Changjiang delta plain with elevation gradient ranging from 2.8 *m* to 3.5 *m*. The climate is subtropical monsoon with an average annual temperature of 16.8 degrees Celsius. Average annual precipitation for the study area is 112 cm and the average annual rainfall days is 137 [26]. Qingpu district is the western suburb of Shanghai and just 20 *km* away from downtown. With the rapid urban sprawl of Shanghai, residential and build-land extended over 100 *km^2^* from 1990 to 2005, which caused the dominant land change of crops land and forest loss [27]. It is representative of Chinese land cover change pattern for the rapid economic development in the last few decades. For the purpose of paying equal attention to farmland protection and development maintenance, the region represents an ideal case study to evaluate semi-automatic land cover classification method applying in numbers of regions of China.

#### Data sources

Three types of data were used for evaluation, including the 1∶100000 land cover maps from two different phases and multispectral images from Environment Satellites 1 (HJ-1). Land covers of 2005 and 2009 were visually interpreted and classified using Landsat TM images and field survey. The land cover maps were produced by the Chinese Academy of Sciences with consistent classification scheme, whose overall accuracy is 95% for all land use classes validated by intensive field surveys. The dataset includes five land cover types: farmland, grassland, forest, residential and construction land, and water. The map of land cover from 2005 was used as prior knowledge for hyperspace analysis and segmentation, while the land cover map of 2009 was used as a reference map for validation. The HJ-1 images of September 22, 2009 were geometrically and radiometrically corrected. Geometric correction was performed by use of 1∶50,000 topographic map and 15 ground control points with a global Root Mean Square Error (RMSE) less than half of spatial resolution (15 m). The HJ-1 images consist of four spectral bands: three visible bands and a near infrared (NIR) band.

### Three-dimensional Feature Space Analysis

**Figure 4:**
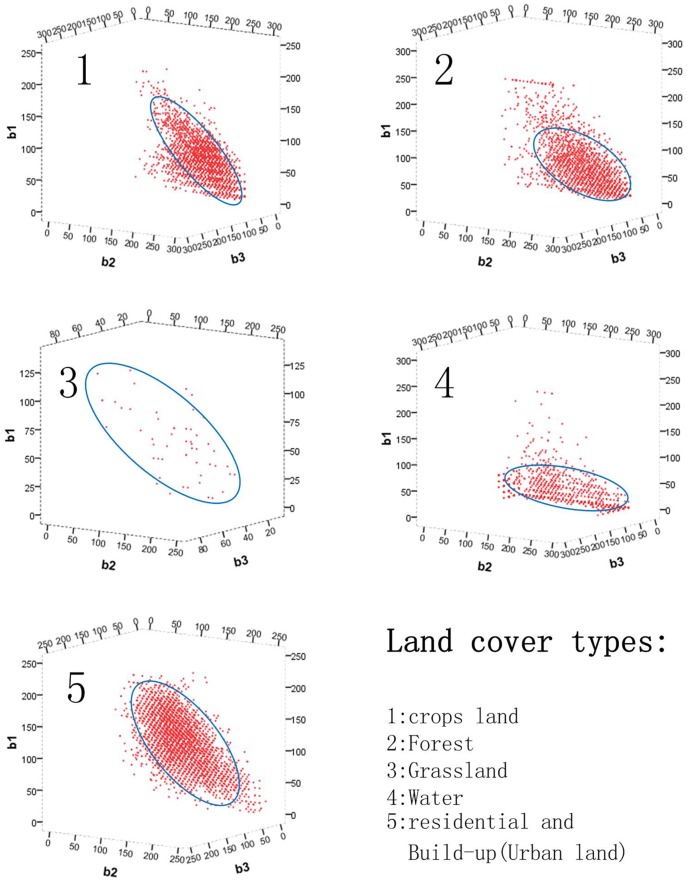
Three-dimensional scatter plots and feature space of five land cover types.

There are five land cover types in the study area: farmland, grassland, forest, water, and residential and construction land (urban space). The spectral Digital Number (DN) values of each type were retrieved with the method described in sections 2.1 and 2.2. Fig. 4 shows the three-dimensional scatter plots and feature space of five types of land cover of interest regions. For this purpose, land cover data derived by visual interpretation of 2005 and HJ-images of September 22, 2009 were adopted.

The results in Fig. 4 show that the spectral DN values of the five land cover types mostly cluster in three-dimensional ellipsoidal spaces. As the sampling regions were retrieved automatically, there are some outliers for each land cover type, which will affect the accuracy of the final classification. In the study area, most of the forest samples were outside of the ellipsoid (Fig. 4.2), accounting for up to 27.6% of the total samples. This may be because the change in forest cover did not follow the ecological rule, due to the high rate of urbanization in Shanghai, causing some large forest patches also to be less ecologically stable. Other land cover types present similar outliers but with fewer samples outside the ellipsoid, which may reduce the accuracy of the classification of forest land. Farmland is the dominant land cover type in the Qingpu District with sufficient samples for our classification method. As shown in Fig. 4.1, there are nearly 14.3% of samples are outside the automatically retrieved three-dimensional ellipsoid space. The area of grassland in the Qingpu District is quite small and spread among the forest and farmland; therefore, there are few grassland samples extracted from the images (Fig. 4.3), which also affects the final classification result. Water, such as rivers and lakes, is the third largest land cover type in the study area. Due to its low reflectivity, water areas are the easiest type to identify from remote sensing images by various other methods. As shown in Fig. 4.4, the DN values of water samples are quite low, and 91.7% pixels of samples in the regions of interest are inside the ellipsoid, ensuring the precision of its classification result. The second major land cover type of residential and construction area (urban land) of study area are of great three-dimensional spatial clustering (Fig. 4.5). Only 4.6% of sample pixels in the region of interest are outliers. In the Qingpu District, urban land is one of the most rapidly expanding land cover types, due to the rapid urbanization of Shanghai City. So within a period of time, the center of a large urban patch is unlikely to be transformed to a different land cover type, and the automatically retrieved samples in this area are well-represented spectrally.

Three-dimensional feature space analysis of the five types of land covers shows that the automatic retrieval of the spectral value of a region of interest can provide support to the successive land cover classification. Fig. 4 shows that samples of forest and grassland in the Qingpu District are less concentrated than the other three land cover types, which limits the accuracy of the final classification result of these two types of land covers. However, the method developed in this study is based on spectral pattern recognition, and the early visual interpretation of land cover data that is used as prior knowledge will offset the spectral bias, ensuring the accuracy of the final results.

### Evaluation

**Figure 5:**
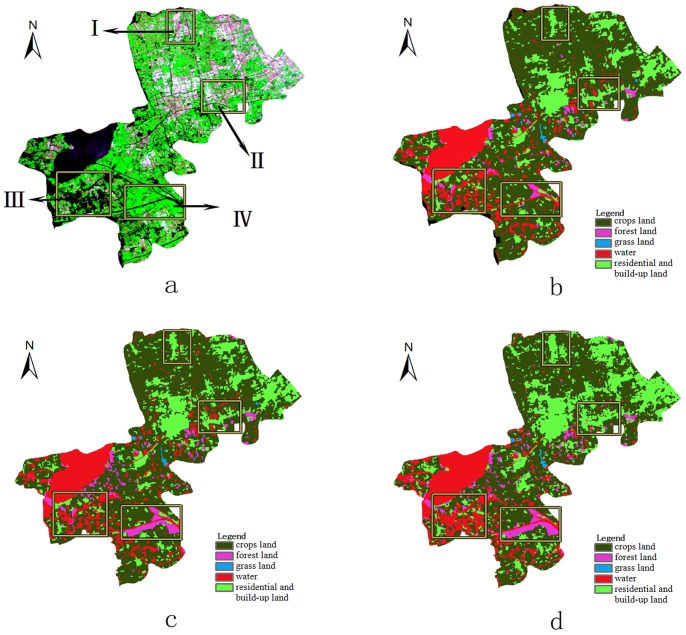
Comparison of land cover classification of Qingpu District. (a) HJ-1 image of 2009.9.22;(b) land cover of 2005;(c) land cover of 2009;(d) land cover classified by the proposed method.

Two types of land cover maps were compared to evaluate the performance of the final classification of the new method: (1) visual interpretation of land cover classes of 2009 is recognized as relatively exact data and (2) the automatically classified land cover of 2009 based on an HJ-1 image following our new method. Fig. 5 illustrates the data sources including the HJ-1 image (Fig. 5-a), and visual interpretation of land cover of 2005 (Fig. 5-b). From the HJ-1 image, the water (black pixels), vegetated land (green pixels) and urban land (other colors pixels) can be easily classified. This determination provides an intuitive basis for the accuracy assessment of classification. Fig. 5-c shows the results of 2009 from implementation of the visual interpretation classification, and Fig. 5-d shows the results using our new method. It should be noted that there are large numbers of pixels (depicted by yellow rectangles) where a significant change in land cover type is found by simply comparing Figs. 5-a and 5-b. Figs. 5-c and 5-d effectively reflect this change from 2005 to 2009.

**Figure 6:**
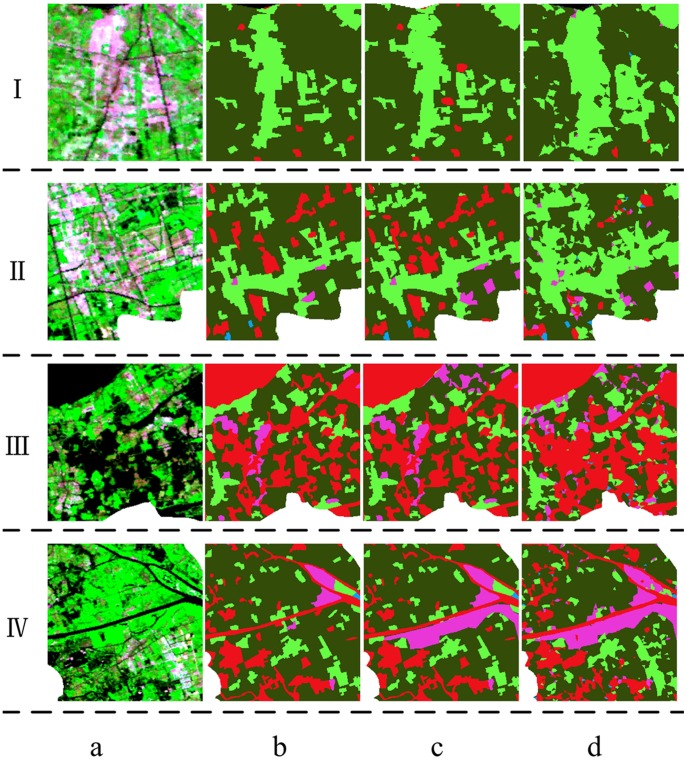
Comparison between the land cover types in the yellow rectangle in Fig. 5. (a) HJ-1 image of 2009.9.22;(b)land cover of 2005;(c)land cover of 2009;(d)land cover classified by the new method.

To better express the performance of this new method in detail, Fig. 6 zooms in the images of the four yellow rectangles in Fig. 5. The results of visual interpretation and our method match the HJ-1 images well, which represents the land cover change during the time interval. In certain ways, Fig. 6-d matches Fig. 6-a better than Fig. 6-c. For Fig. 6-c is visual interpretation classified land cover data based on Landsat TM images of whole year of 2009. The borders of land cover patches in Fig. 6-c are sharper than those derived using the new method for the different implemented algorithms, which is consistent with the actual land cover of the Earth’s surface. In this study, the 2009 visual interpretation data were considered to be the reference for assessing the accuracy of the results of the new method.

### Assessment of Overall Spatial Accuracy

Fig. 5 and Fig. 6 show that the result of our method closely matches the HJ-1 image and visually interpreted land cover in intuitive view. To quantitatively assess the spatial accuracy of classification land cover from the simple method, Table 1 provides statistics of the five land cover classes of the Qingpu District.

**Table 1:** Statistics of five land cover classes of the three classification results.

		Crops land	Grass land	Forest land	Water	residential and build-up land
visual interpretation land cover of 2005	area(km^2^)	425.7	2.1	14.3	110.3	117.9
	proportion(%)	63.5	0.3	2.1	16.5	17.6
visual interpretation land cover of 2009	area(km^2^)	391.2	2.1	26.7	108.6	141.0
	proportion(%)	58.4	0.3	4.0	16.2	21.1
Land cover of new method	area(km^2^)	360.9	3.0	29.9	113.8	163.0
	proportion(%)	53.8	0.4	4.5	17.0	24.3

The area of five land cover classes for 2009 derived from visual interpretation and our method were compared, and there was little bias between two sets of results. According to the visually interpreted data, the large areas of changed land cover in the Qingpu District from 2005 to 2009 are farmland and residential and construction land. The area proportion of farmland to whole region declined from 63.5% to 58.4%, whereas the proportion of residential and construction land grew from 17.6% to 21.1%. It was consistent with the rapid urbanization of Shanghai, which could be interpreted as the urban land overtaking farmland. The proportions of farmland and residential and construction land using our new method are 53.8% and 24.3%, respectively. Compared to visually interpreted results, the errors of the two land cover classifications are 5.4% and 3.2%. The other three land cover classes (grassland, forest and water) changed less from 2005 to 2009. The biases in proportion of the three land covers derived from our automatic classification method are 0.1, 0.5 and 0.8 compared to the visually interpreted land cover of 2009.

### Pixel-by-pixel Analysis of the Agreement of Results

For the next phase of analysis, each pixel of the land cover classification maps using the new method was compared to its counterpart in the visually interpreted land cover map of 2009 on a pixel-by-pixel basis. Statistics on the accuracy of the findings are summarized by presenting Cohen’s Kappa [28]. Although overall accuracy is often used as a standard indicator of map quality, many contend that Kappa provides a better overall measure because it also incorporates information on the errors of omission and commission [29], [30].

**Table 2:** Confusion matrix of two classification algorithms of Qingpu District, 2009.

		New method					** 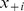 **
		Crops land	Forest land	Grass land	Water	residential and build-up land	
visual interpretationclassification	Crops land	381111	1648	1247	22685	24942	431633
	Forest land	2696	22121	14	2592	2024	29447
	Grass land	328	116	1751	73	77	2345
	Water	8912	4947	308	98505	7182	119854
	residential andbuild-up land	7883	4367	17	2484	141340	156091
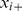		400930	33199	3337	126339	175565	739370

Kappa value (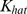 = 0.79).

Table 2 shows the land cover confusion matrix of results of our method and visual interpretation classification of 2009. The overall accuracy is 87.2%, which can be calculated from Table 2. According to analysis of Table 2, the misclassification rates of land cover classes of forest and grassland are 24.9% and 25.3%, respectively, which are higher than the misclassification rates of the other three types of land cover classes. The reason for this can be found in the automatic retrieval spectral values of the study region described in section 3.2. Three-dimensional scatter plots and the feature space of forest and grassland (Figs. 4.2 and 4.3) show that the two land cover classes are not as representative as the other three land cover types. However, forest and grassland occupied small proportions of the entire Qingpu District, which reduces the inaccurate effect on the map agreement. The other relatively map omission and commission error existed among crops land, water and residential and build-up land. In Table 2, some pixels of crops land were classified to water and residential and build-up land. They were mainly caused by the land cover classification system, especially crops land (including two sub- categories of paddy field and dry farming field) and residential and build-up(including three sub-categories of urban land, rural residential and other build-up land). Urban land includes landscape such as water for rest, may be classified to categories of residential and build-up in visual interpretation classification. It will caused the mis-classification between water and residential and build-up land. Rural residential land always scatters inside of large area of crops land in China, which will increase map omission and commission error of these two land covers. In addition, paddy field,normally flood irrigated in China, may present similar spectral signature to water, which will introduce mis-classification between crops land and water. Acknowledging these spectral limitations, the accuracy of classification result of Qingpu distribution is acceptable.

The Kappa value(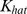 = 0.79) of the two land cover classification maps of 2009 are calculated based on values in Table 2. Blackman and Landis [31], [32] graded Kappa value between 0 and 1 for the analysis of map agreement, which has become the standard for the agreement of maps in practice. The Kappa value for the results of our method and the validation visual interpretation classification of 2009 is 0.79, which means the two maps agree well (a Kappa value between 0.61 and 0.8 is considered to be in good agreement [31], [32]). The proposed approach gives acceptable land cover classification in the Qingpu District. Furthermore, residential and construction land (urban land) has the best precision of the five land cover types, which indicates that this method is sensitive to changes in residential and construction land.

We also tested the accuracy of methodology for three different regions chosen based on their degrees of land cover change. These results show that the proposed method performs well in regions with normal-to-high rates of land cover change, especially droved by rapid urban development. While its accuracy is slightly lower in regions with little land cover change, such as natural conservation areas. There is mainly result from changed rules described in step 2.4. The land cover of a natural conservation area is relatively stable, and the small land cover change areas are natural affected without rules which will increase error of ultimate result of land cover classification. Some following work, such as study of the zoning in land cover change and a better-planned rules repository, may be useful in improving the performance of the method in the future.

### Conclusion

Land cover data are important for research on global environmental change [32]. Remote sensing images are important data sources for land cover mapping [33]; however, the existing methods for land cover classification based on remote sensing images are not sufficiently flexible or effective [34], [35]. Based on early land cover data used for change detection and the assumption that land cover changes are ecologically stable and in accordance with the changing rules, a simple but robust automatic land cover classification method was proposed. The satellite images can be automatically classified using only the prior land cover map and current images with less human interaction or interpretation. By applying the newly developed method to the Qingpu District of Shanghai and by comparing the results with a visually interpreted land cover map, it was found that the two land cover maps closely agree with each other, and the new method is appropriate for land cover classification. The new approach demonstrates the following three advantages over existing methods: (1) it automatically obtains training samples with Geography Information System (GIS) and statistical technology; (2) it uses prior land cover maps as background knowledge to guarantee the accuracy of the final classification result; and (3) it revises the classification result based on the changed land cover rule in certain areas, which avoids errors originating from relying solely on the spectral pattern of remotely sensed images, and it reflects societal influence on land cover change.

The approach proposed in the paper is similar with state-of-the-art Support Vector Data Description (SVDD) method for they both can learn from contaminated training data (both inners and outliers) in the feature space. However, we provide different solutions to this problem. In our paper, the results are competitive for the feature space was established using the prior land cover knowledge and current images, while the SVDD using difference among images of different times.

Whereas, there are four points that need to be mentioned when implementing this method: (1) the classification results would be more accurate if the early land cover data were more precise; (2) a long time interval between the early land cover data and the classification images is not advisable, and results of our experiments suggest that a data gap of no more than five years is acceptable for China; (3) the changed land cover rule needs to be constructed based on the land cover policies of the study area and the expert’s knowledge; and (4) images classification of area with irregular land changes, especially the area of changes mainly happens in the domain(e.g., commercially managed forests, type changed crops), will introduce challenges to the method. When training samples collected away from edges (where land cover is more likely ecologically stabile) are less pure or mixed pixels of specific land cover categories, error will be caused in final mapping.
